# A Peripheral Immune Signature of Labor Induction

**DOI:** 10.3389/fimmu.2021.725989

**Published:** 2021-09-09

**Authors:** Kazuo Ando, Julien J. Hédou, Dorien Feyaerts, Xiaoyuan Han, Edward A. Ganio, Eileen S. Tsai, Laura S. Peterson, Franck Verdonk, Amy S. Tsai, Ivana Marić, Ronald J. Wong, Martin S. Angst, Nima Aghaeepour, David K. Stevenson, Yair J. Blumenfeld, Pervez Sultan, Brendan Carvalho, Ina A. Stelzer, Brice Gaudillière

**Affiliations:** ^1^Department of Anesthesiology, Perioperative and Pain Medicine, Stanford University School of Medicine, Stanford, CA, United States; ^2^Department of Biomedical Sciences, University of the Pacific, Arthur A. Dugoni School of Dentistry, San Francisco, CA, United States; ^3^Division of Neonatal and Developmental Medicine, Department of Pediatrics, Stanford University School of Medicine, Stanford, CA, United States; ^4^Department of Biomedical Data Science, Stanford University School of Medicine, Stanford, CA, United States; ^5^Department of Obstetrics and Gynecology, Stanford University School of Medicine, Stanford, CA, United States

**Keywords:** labor, pregnancy, parturition, induction of labor, systems immunology, mass cytometry (CyTOF), machine learning

## Abstract

Approximately 1 in 4 pregnant women in the United States undergo labor induction. The onset and establishment of labor, particularly induced labor, is a complex and dynamic process influenced by multiple endocrine, inflammatory, and mechanical factors as well as obstetric and pharmacological interventions. The duration from labor induction to the onset of active labor remains unpredictable. Moreover, prolonged labor is associated with severe complications for the mother and her offspring, most importantly chorioamnionitis, uterine atony, and postpartum hemorrhage. While maternal immune system adaptations that are critical for the maintenance of a healthy pregnancy have been previously characterized, the role of the immune system during the establishment of labor is poorly understood. Understanding maternal immune adaptations during labor initiation can have important ramifications for predicting successful labor induction and labor complications in both induced and spontaneous types of labor. The aim of this study was to characterize labor-associated maternal immune system dynamics from labor induction to the start of active labor. Serial blood samples from fifteen participants were collected immediately prior to labor induction (baseline) and during the latent phase until the start of active labor. Using high-dimensional mass cytometry, a total of 1,059 single-cell immune features were extracted from each sample. A multivariate machine-learning method was employed to characterize the dynamic changes of the maternal immune system after labor induction until the establishment of active labor. A cross-validated linear sparse regression model (least absolute shrinkage and selection operator, LASSO) predicted the minutes since induction of labor with high accuracy (R = 0.86, p = 6.7e-15, RMSE = 277 min). Immune features most informative for the model included STAT5 signaling in central memory CD8^+^ T cells and pro-inflammatory STAT3 signaling responses across multiple adaptive and innate immune cell subsets. Our study reports a peripheral immune signature of labor induction, and provides important insights into biological mechanisms that may ultimately predict labor induction success as well as complications, thereby facilitating clinical decision-making to improve maternal and fetal well-being.

## 1 Introduction

Induction of labor is the pharmacological initiation of cervical change and uterine contractions before their spontaneous onset in the presence or absence of ruptured membranes (1). While induction of labor is typically recommended in the setting of deteriorating maternal and/or fetal status (2–4), a recent prospective randomized controlled trial of elective labor induction after 39 weeks’ gestation showed the safety and benefit of induction, including lower rates of cesarean delivery, even in the absence of a medical indication (5). In the United States, labor inductions are increasingly being performed and now constitute between 25% to 50% of all deliveries (6, 7). Unfortunately, the time interval between labor induction, the establishment of latent labor (defined as regular contractions leading to cervical changes from 0 to 6 cm) and the onset of active labor (defined as cervical dilatation ≥ 6 cm with regular uterine contractions ≤ 3 min apart) remains unpredictable. Similarly, induction complications associated with failure of successful labor initiation, prolonged labor progression, subsequent labor arrest, development of chorioamnionitis or failed induction resulting in cesarean delivery (7–9), are difficult to predict. Prolonged labor is associated with severe complications and morbidity for the mother and her offspring, most importantly chorioamnionitis, uterine atony and postpartum hemorrhage (4). A better understanding of the biological events temporally associated with the progression from labor induction until the establishment of active labor is much needed to identify predictive biomarkers of successful labor induction and prevent clinical complications resulting from failed induction.

The biology underlying the onset and establishment of labor remains incompletely understood. Labor is characterized by changes of the fetomaternal physiology including infiltration of immune cells into fetal membranes and the placenta (10–12), endocrine adaptations (13–15), rupture of fetal membranes (16), cervical dilation, and augmentation of uterine contractility (17), culminating in the delivery of the fetus. The local environment at the time of active labor is inflammatory (12, 18–21), which is echoed and detectable in maternal circulating immune cells [including activation of neutrophils, increased frequencies of CD56^+^CD16^+^ natural killer (NK) cells, and increased inflammatory cytokines (22, 23)]. However, the biological determinants that contribute to the variability in labor induction development from onset of labor to active labor are currently largely unknown.

In this study, we performed an in-depth immune profiling of peripheral blood samples collected between the induction of labor to the establishment of active labor. We employed a high-dimensional single-cell immunoassay (mass cytometry), which has previously enabled tracking peripheral immune cell distribution and functional adaptations in maternal blood over the course of pregnancy (24–28), and paired it with pertinent computational algorithms to identify a maternal immune signature predictive of the time since induction of labor.

## 2 Results

### 2.1 Assessment of the Maternal Immunome After the Induction of Labor

Fifteen pregnant women receiving antepartum care at Lucile Packard Children’s Hospital (Stanford, CA, USA), were enrolled in their third trimester of pregnancy. Study participants included were women with term (> 37 weeks’ gestational age) singleton pregnancies who underwent planned induction of labor for reasons unrelated to infection or inflammatory conditions. All study participants presented with intact membranes and had no regular uterine contractions at the time of induction. Patient demographics including reason for and method of labor induction are shown in **Table 1**.

**Table 1:** Demographic data of the study cohort.

Pregnancy Characteristics	N=15, n=48*	Percentage or Median (Interquartile Range)
Age (years)	15	33 (29, 34)
Body mass index (BMI) 3^rd^ trimester (kg/m^2^)	13	33 (27, 35)
Gestational age at delivery, all (weeks)	15	39.6 (39.1, 41)
Gravity	15	1.0 (1.0, 2.0)
Parity (% nulliparous)	15	13 (86.7)
Fetal sex (% female)	15	12 (80)
Birthweight (g)	15	3320 (3049, 3556)
5-min Apgar score	15	9 (8.75, 9)
**Race**		
Asian	7	46.7
African-American	0	0
Native Hawaiian/Pacific Islander	1	0.066
Middle Eastern	0	0
White	6	40
Two or more races	1	0.066
**Ethnicity**		
Hispanic	4	26.7
Non-Hispanic	11	73.3
**Labor and Delivery**		
Induction of labor	15	100
**Indication**		
Beyond estimated day of delivery	7	46.7
Elective	1	0.066
History of chronic hypertension	3	20
Advanced maternal age	1	0.066
Other	3	20
**Initial Medication**		
Misoprostol	11	73.3
Oxytocin	3	20
Oxytocin and cervical balloon	1	0.066
Cervidil	1	0.066
**Time elapsed since induction at sampling (in min)**		
1 hr post-induction	15	65 (95% CI 5)
Regular uterine contractions	7	410 (95% CI 329)
Cervical change	3	560 (95% CI 74)
Start of active labor	8	1277 (95% CI 276)
**Labor Complications**	6	40
Arrest of dilation and cesarean section	3	20
Arrest of descent after full cervical dilation and cesarean delivery	1	6.7
Chorioamnionitis, arrest of dilation, and cesarean delivery	1	6.7
Chorioamnionitis and vaginal delivery	1	6.7
**Mode of Delivery**		
Vaginal delivery	10	66.7
Cesarean delivery	5	33.3
**Comorbidity**		
Gestational diabetes	1	0.066
Polycystic ovary syndrome	0	0
Gestational hypertension	2	13.4
Preeclampsia without severe features	1	0.066
Anxiety/depression	1	0.066
History of preterm birth/Progesterone treatment	0	0

*N, number of patients; n, total number of samples.

**Figure 1:**
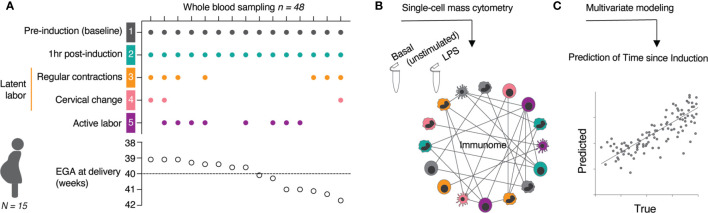
Experimental workflow and analytical approach. **(A)** Whole blood samples were collected at indicated time points (T1 to T5) from 15 women with term pregnancies undergoing induction of labor. **(B)** A mass cytometry immune-assay was employed to measure the frequency, and single-cell intracellular signaling activities in all major immune cell populations at basal level (unstimulated) and after 15 min stimulation with LPS. **(C)** The high-dimensional data set was used in a multivariate modeling approach to predict the time since induction (TSI) from the correlation relationship between all immune features across sampling time points. See also **Table 1**.

For each study participant, serial whole blood samples were obtained at 5 timepoints (**Figure 1A**): The first sample was obtained immediately prior to induction (baseline) (T1, gray). The next three timepoints were obtained during the establishment of the latent phase of labor, including 1 hr after induction (no cervical change since admission) (T2, teal), at the start of regular uterine contractions (contractions ≤ 3 min apart) (T3, yellow), and at first cervical change since admission (T4, pink). The last timepoint (T5, purple) was obtained at commencement of active labor (defined as cervical dilation ≥ 6 cm with regular uterine contractions ≤ 3 min apart). The approach leveraged the interindividual variabilities in sample collection time to define a continuous variable, time since induction (TSI), which describes the difference (in min) between the point of induction and the time of sampling. Using a 46-parameter mass cytometry assay (**Supplementary Table 1**), a total of 1,059 single-cell immune features were extracted from each sample including the frequencies of 46 immune cell subsets representing major innate and adaptive populations, endogenous intracellular activities (e.g., phosphorylation state) of 11 signaling proteins, and capacities of each cell subset to respond to a receptor-specific immune challenge [lipopolysaccharide (LPS)] (**Figure 1B**). The interrelatedness of immune feature trajectories justified the use of a multivariate approach to identify biologically relevant components of the maternal immunome predictive of the TSI (**Figure 1C**).

### 2.2 Distinct Peripheral Immune Responses Demarcate the Transitions to Latent and Active Labor

**Figure 2:**
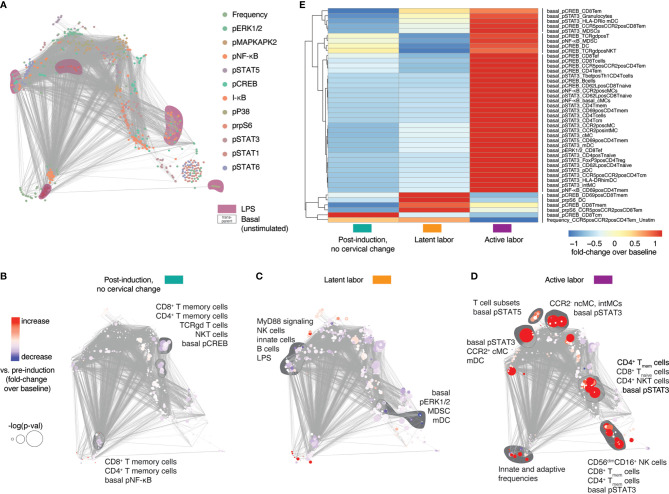
Systemic immune responses in maternal blood after labor induction. **(A)** Correlation network showing the relationships between immune features within and across mass cytometry data categories. Features and communities are annotated based on stimulation condition, and functional marker. **(B–D)** Correlation networks depicting immune feature changes 1 hr post-induction (no cervical change since admission, T2, teal), in the latent phase of labor (T3, yellow), and with active labor (T5, purple), compared to baseline (pre-induction) (T1). Node color indicates increase/decrease (red/blue) compared to baseline (T1). Node size represents p-value (adjusted for multiple comparisons) associated with paired **(B)** and unpaired **(C, D)** T tests compared to baseline (univariate comparisons). **(E)** Heatmap showing Z-scored fold change feature behavior across time points [post-induction (T2, teal), in the latent phase of labor (T3, yellow), and with active labor (T5, purple)] compared to baseline (T1). Shown are features significantly different from baseline (p < 0.05, Wilcoxon signed-rank test for T1 vs. T2 or rank-sum test for T1 vs. T3-5). See also **Supplementary Figure 1** and **Supplementary Table 2**.

We first determined whether peripheral immune responses differentiated two clinically important transitions during labor progression in all fifteen study participants (primary outcome): the transition between labor induction and the onset of latent labor, and the transition between latent labor and active labor. Peripheral immune cell frequencies and intracellular signaling activities assessed at time point T1 to T5 were integrated and visualized on a correlation network (**Figure 2A**). Immune features segregated into highly correlated communities highlighting the relationship between immune features progressing synchronously with labor progression (**Figures 2B–D**, **Supplementary Figure 1**, **Supplementary Table 2**). When compared to pre-induction baseline, cell-type specific changes in immune signaling activities were first observed as early as 1 hr post-induction (**Figure 2B**), with the transition to latent labor (**Figure 2C**), and with the transition to active labor (**Figure 2D**). Specifically, downregulation of CREB signaling in CD4 and CD8 memory T-cell populations was observed 1-hr post-induction and during latent labor compared to baseline (**Figure 2B**). The transition to latent labor was paralleled by the downregulation of multiple elements of the MyD88 signaling pathway in innate immune cells (**Figure 2C**). In contrast, the transition to active labor was characterized by an immune-system-wide activation of pro-inflammatory signaling responses, including STAT3 signaling in multiple innate and adaptive cell subsets [including classical monocytes (cMC), myeloid dendritic cells (mDC), naïve and memory CD4 and CD8 T cells, and NK cells], STAT5 in several CD8 and CD4 T-cell subsets, and increased frequencies of e.g., circulating granulocytes (**Figure 2D**).

Immune features that differed (p < 0.05, Wilcoxon signed-rank test for T1 vs. T2 or rank-sum test for T1 vs. T3-5) from the pre-induction baseline (T1) were summarized on a heatmap (**Figure 2E**) which highlighted marked differences in immune responses before (1 hr post induction, no cervical change since admission, teal), and after the onset of latent labor (yellow), as well as with the establishment of active labor (purple). One particular example includes changes in STAT3 and CREB signaling in several CD8 and CD4 T-cell subsets (**Figure 2E**). The results suggest that the clinical transitions between labor induction, latent labor and active labor are echoed by peripheral immune responses that can be detected in the maternal blood.

### 2.3 Multivariate Modeling of Maternal Immune Cell Dynamics After Induction of Labor

**Figure 3:**
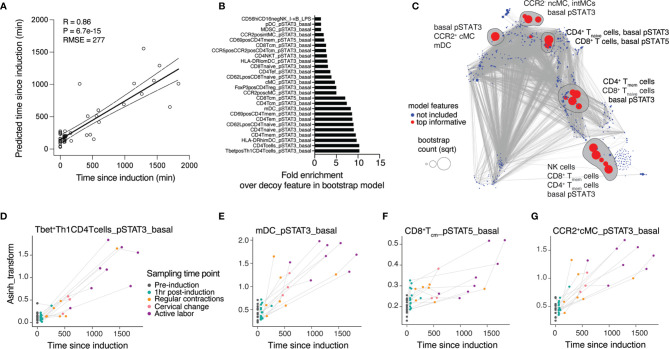
A regression model accurately predicts dynamic changes of the maternal immune response throughout the latent phase of labor. **(A)** Regression of predicted vs. true time since induction (TSI) derived from the LASSO model (Spearman R = 0.86, cross-validation, p-value = 6.7e-15, RMSE = 277 min, N = 15 patients). **(B)** Top informative feature ranking derived from occurrences in a bootstrap analysis with 1,000 iterations. **(C)** Correlation network of cross-validated LASSO model predicting TSI at time of sampling. Red color highlights features top informative for the prediction model. Blue features did not inform the model and were not included. Dot size indicates the bootstrap count per feature (square-rooted). Top model features selected by bootstrap analyses are circled. **(D–G)** Dynamics of individual top model features across time points during latent labor. See also **Supplementary Figure 2** and **Supplementary Table 3**.

The analysis of peripheral immune cell responses at individual timepoints provided insight into maternal immune system variations associated with predefined clinical transitions. In order to integrate these insights describing one predefined state of labor at a time into a continuous model of immune response changes over time since labor induction, we employed a multivariate approach that leveraged the variability in sample collection time to build an immunological model predicting the time since induction of labor (**Figures 2A–D**). In this approach, a linear sparse regression model, LASSO (least absolute shrinkage and selection operator) model was built from all immunological features of all fifteen patients to predict the TSI variable (Pearson R = 0.86, p = 6.7e-15, root-mean-square-error (RMSE) = 277 min) (**Figure 3A**). Statistical significance was established using leave-one-patient-out cross-validation strategy to estimate the model performance, thereby accounting for the non-independence of samples collected from the same patient (see Methods).

To facilitate the biological interpretation of the model, we focused on the model features that most robustly contributed to the prediction of the TSI (**Figure 3B**). We identified 26 features that occurred at a higher frequency than any artificial decoy features across models generated in an iterative bootstrap analysis (**Supplementary Table 3**, see Methods). These top-informative features were projected onto the correlation network generated from all features (**Figure 3C**). Examining the trajectories of individual model features over time (**Figures 3D–G**) revealed that basal pro-inflammatory signaling dominantly informed the TSI prediction. Specifically, two members of Jak-STAT signaling pathways, STAT3 (including in Tbet^+^ Th1 CD4 T cells, mDC, CCR2^+^ cMC) and STAT5 (in CD8 T_cm_ cells), evolved in a coordinated dynamic pattern between the time of labor induction and the time of establishment of active labor (**Figures 3D–G** and **Supplementary Figure 2**).

In summary, our assessment of maternal circulating factors in the peripheral blood provided a prediction model of the time from induction until the start of active labor, unraveling a systemic immune signature strongly associated with the progression of labor towards the active phase of labor (timepoint T5).

### 2.4 Immune Signatures of Labor Induction Diverge Between Uncomplicated and Complicated Labor

Six study participants in our cohort suffered from complications of labor induction, allowing us to explore whether the systemic immune signature after labor induction differed between women with uncomplicated and complicated labor. For purposes of post-hoc exploratory analyses of our study cohort, we defined a complicated labor as any labor indication that was associated with labor arrest or chorioamnionitis. Both conditions are associated with a prolongation of labor, suggesting that these complicated labors are related to the variable of our prediction model, ‘time since induction of labor’ (29, 30). Among the 6 patients with complications, three experienced arrest of labor and subsequently required intrapartum cesarean delivery (n = 3; cervical dilation at time of cesearean delivery was 4, 5, and 6 cm), one patient experienced arrest of descent after full cervical dilation (n = 1), while two patients developed chorioamnionitis, delivering either vaginally (n = 1) or experiencing arrest of labor with subsequent requirement for cesarean delivery (n = 1; cervical dilation at time of cesarean delivery was 5 cm) (**Table 1**).

**Figure 4:**
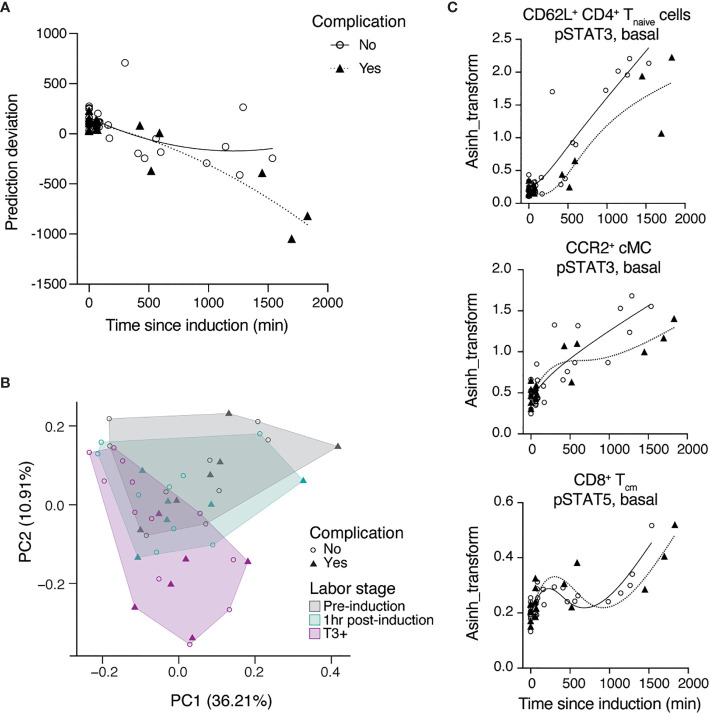
Differences in immune trajectories between complicated and uncomplicated labor. **(A)** Prediction deviation from the true TSI [residual error (predicted – true TSI)] stratified by absence (open circles) or presence (triangles) of labor complications. **(B)** PCA analysis identified two components that explain 36.2% (PC1) and 10.9% (PC2) of the variation across phases of latent labor, where complicated labor (triangles) majorly clusters along the axis of PC2. **(C)** Representative features among the top predictive features which follow different trajectories in patients with (triangles) or without (open circles) complications after the induction of labor (lines represent splines with 4 knots). See also **Supplementary Table 3**.

An exploratory analysis was performed to determine whether systemic immune signatures observed before the establishment of active labor could signal a complication of labor induction. The median time from labor induction to active labor was 985 min (16.4 hrs) for the 9 women who underwent uncomplicated inductions with vaginal delivery and 1767 min (29.4 hrs) for 2 women among the group of complicated labor who did advance to active labor. A post-hoc analysis comparing women with uncomplicated (associated with an uncomplicated vaginal delivery) vs. complicated induction (associated with labor arrest or chorioamnionitis) revealed that the prediction model performed with higher accuracy in uncomplicated (RMSE = 220 min) compared to complicated (RMSE = 353 min) labor induction. Plotting the residual errors of predicted over true time since induction showed increased uncertainty in prediction for complicated labor, which was specifically pronounced with prolonged time after induction until active labor (T3 to T5) (**Figure 4A**). The difference in error of prediction indicated a systemic bias in the immune signature of complicated labor compared to those patients with uncomplicated labor inductions. The differences between immune signatures in women with uncomplicated vs. complicated labor were further evaluated using a principal component analysis (PCA) (**Figure 4B**). The analysis revealed that while the immune signature of labor was captured in Principal Component PC2 (R = -0.72, p = 1e-8), PC2 correlated better [R = -0.77 (complicated) vs. R = -0.69 (uncomplicated)] with the immune signature of labor for complicated labor, thus indicating that the variance in these samples can be explained by complication-specific deviations. Consistent with the PCA results, different dynamics were observed for the most informative features of the multivariate model of the immune signature of labor induction (including Jak-STAT signaling) in patients with uncomplicated vs. complicated labor (**Figure 4C**). Specifically, the changes of STAT3 signaling in CD4 T cells (p = 0.02) and cMC (p = 0.28), as well as STAT5 in CD8 T_cm_ cells (p = 0.84) were delayed, i.e., occurred later after induction, in patients with complicated labor compared to patients with uncomplicated labor (**Supplementary Table 3**). In summary, the data suggests that a lag in the development of immune responses in the early phase after labor inductions may be associated with subsequent complications as labor progresses.

## 3 Discussion

We employed a single-cell mass cytometry immunoassay to comprehensively characterize the dynamic changes in maternal immune cell distribution and signaling responses between the time of induction of labor and the establishment of active labor. Analysis of the high-dimensional data identified a multivariate model that accurately predicted the time since induction of labor, thus demarcating a peripheral immune signature of latent phase progression detectable in the maternal blood. Examination of individual model components revealed the cell-type specific activation of pro-inflammatory signaling responses with progressing labor, including the activation of the STAT3 and STAT5 signaling pathways across several innate and adaptive immune cell subsets. Remarkably, differential immune cell response dynamics were observed in a posthoc analysis comparing women with uncomplicated vs. complicated labor induction providing a promising experimental framework for the identification of blood-based immunological markers for the successful induction of labor.

Labor is initiated upon myometrial activation and associated cervical change. It is well described that myometrial responsiveness to uterotonic agents, such as prostaglandins and oxytocin, is mediated by increased expression of labor-promoting genes to establish synchronous myometrial contractions (31). Despite high levels of circulating labor-suppressing progesterone at term, the myometrium undergoes a functional progesterone withdrawal at onset of labor, mediated by the repression of type-B progesterone receptor (PR-B) functions by PR-A. Strikingly, this repressive activity of PR-A has been shown to be induced by pro-inflammatory conditions *in vitro*, linking inflammation with labor onset (32).

We observed pro-inflammatory features across innate and adaptive immune cell populations at the beginning of active labor, most prominently in the STAT3 pathway, which is consistent with previous studies reporting the systemic release of its extracellular stimuli with evolving labor (20, 33). Importantly, in tissues such as the endometrium and breast cancer cells, STAT3 has been shown to specifically bind to PR-A (34), and co-activate its transcriptional functions (35). While this mechanism may contribute to enhancing myometrial contractility, STAT3 binding to PR-A in multiple immune cells may also support well-described immune cell migration into fetal membranes and the decidua during labor by interfering with progesterone responsiveness (20, 36). STAT3 is an acute-phase response factor and is rapidly activated by a range of cytokines with pro- (IL-6, IFNs, or TNF-α), and anti-inflammatory (IL-10) function (37). It likely contributes to promoting labor as the prominent intracellular downstream target of IL-6, a known labor initiator (38, 39), by boosting cortisol-induced prostaglandin production in the amniotic membranes (40). Secreted by immune cells and the myometrium, IL-6 stimulates a feed-forward loop to enhance chemotaxis of monocytes and T cells to the choriodecidua and promote inflammation, which is considered a key step for labor initiation (12). Previously reported enhanced IL-6 gene expression in circulating leukocytes during active labor match our observations of active-labor associated STAT3 signaling (23, 36). Interestingly, immune responses were broadly dampened after labor induction and during latent labor, in comparison to pre-induction, while robust activation of pro-inflammatory signaling responses was observed with the transition to active labor. The results suggest that the peripheral inflammatory response is modulated in parallel with myometrial activation, supporting the notion that several corroborating mechanisms contribute to labor initiation (17).

While risk factors for failure of labor to progress are described, proxies to anticipate failed or complicated labor during the active phase of the first stage of labor after labor induction that are measurable during the latent phase of labor are lacking (41, 42). It is known that a longer duration of the latent phase (> 15 hrs) is associated with increased risk of subsequent cesarean delivery and complications such as maternal hemorrhage and chorioamnionitis (4). We report an association between a delay in the evolution of the peripheral immune signature during the latent phase of labor, and subsequent labor arrest and chorioamnionitis. In our cohort, this delay appeared most pronounced at T5 (start of active labor), indicating that the immune profile indeed follows the clinically-relevant phases of labor between initiation of labor and active labor. This delay may, pending future studies, function as a potential indicator of ensuing labor arrest and associated complications.

Our study has several limitations. The number of samples in our study cohort was sufficient to analyze the immune profile of successful labor induction, yet it lacked statistical power to stratify labor progression based on absence or presence of complications and to stratify patients according to other important clinical factors. For example, we acknowledge that the likelihood of failed induction of labor may be influenced by other maternal demographic (ethnicity, age, weight, height), obstetric (parity, placental insufficiency, multiple gestation, prelabor rupture of membranes, gestational age, cervical status upon commencing induction and method of induction), neonatal (fetal weight) and medical factors (43–47). Much of the literature surrounding failed induction of labor use different or poorly defined endpoints such as failure to delivery vaginally, not achieving vaginal delivery within 24 hrs, or not entering the active phase of labor. However, our work now justifies to design studies in larger cohorts with the aim of identifying predictive immune markers of failed induction of labor prior to commencement of active labor (9, 45–48). Potential future clinical studies should also include parallel analyses of choriodecidual material to help understand local immune responses. Lastly, studying labor in patients for whom labor was induced, precludes conclusions about the progression of spontaneous labor occurring without medical intervention. Further studies are needed to elucidate immune signatures beyond the time point of active labor, until delivery of the fetus. Generalization studies are also required in different ethnic groups and patient populations from different geographical locations using the model developed in this cohort, in order to further validate our findings, and evaluate model performance. Ultimately until equipment used in this study becomes more widely available to institutions, the applicability of such prediction modeling may be limited to academic centers and is likely to also be limited by the costs and time taken to perform such functional assays.

In summary, we successfully derived an immune signature of successful labor induction and progression in peripheral maternal blood. Specific variation of this signature, particularly during the latent phase of labor, may allow differentiating between successful and failed or complicated labor induction, which in turn may facilitate clinical decision-making to improve maternal and fetal well-being.

## 4 Materials and Methods

### 4.1 Study Design and Sample Collection

The aim of this observational study was to determine whether a precise chronology of immunologic adaptations related to labor is detectable in peripheral immune cell phenotype and functional changes analyzed serially during labor, starting prior to medical induction of labor. The study was conducted at the Lucile Packard Children’s Hospital (Stanford, CA, USA). The study was approved by the Institutional Review Board (Approval ID: 44576), and all participants signed an informed consent. Healthy pregnant women receiving routine antepartum care were eligible for the study if they were within 18 to 50 years of age, body mass index (BMI) < 40, in their 39 to 41 gestational week of a singleton pregnancy as determined by their clinician using last menstrual period and ultrasound estimates of gestational age, and had no co-morbidities or concurrent medication that may have a confounding immune-modifying effect (e.g., autoimmune disease, gestational or longstanding diabetes requiring insulin or other antigylcemic agents), no history of any illicit drug use, and no significant fetal anomalies. In total, 15 women were recruited to meet the pre-determined sample size required for sufficient power in this longitudinal study. Participants with healthy, singleton pregnancies, who required an induction of labor or elected induction of labor were included in the analysis. Demographics, pregnancy characteristics, reasons for induction, and comorbidities for the participants included in the analysis are summarized in **Table 1**.

After labor induction, participants were followed through the establishment of latent labor (defined as regular contractions leading to cervical changes from 0 to 6 cm) until the onset of active labor (defined as cervical dilatation ≥ 6 cm with regular uterine contractions ≤ 3 min apart). For each study participant, whole venous blood was obtained at 5 timepoints. The first sample was obtained (T1) immediately prior to induction (baseline). The next three timepoints were obtained during the establishment of latent phase of labor, including (T2) 1 hr after induction (no cervical change since admission), (T3) at the start of regular uterine contractions (less than every 3 min), and (T4) at first cervical change since admission. The last timepoint (T5) was obtained at commencement of active labor. The variability in labor progression (e.g., immediate advancement to active labor) precluded sample collection during pre-defined labor stages from individual women.

### 4.2 Mass Cytometry

#### 4.2.1 *Ex Vivo* Whole-Blood Immuno-Assay

Whole blood was collected from study subjects and processed within 60 min after blood draw. Individual aliquots were stimulated for 15 min at 37°C with LPS (1 ug/mL, InvivoGen, San Diego, CA), or left unstimulated. Samples were processed using a standardized protocol for fixing with proteomic stabilizer (SMART TUBE, Inc., San Carlos, CA) and stored at -80 °C until further processing.

#### 4.2.2 Sample Barcoding and Minimization of Experimental Batch Effect

To minimize the effect of experimental variability on mass cytometry measurements between serially collected samples, samples corresponding to the entire time series collected from one woman were processed, barcoded, pooled, stained, and ran simultaneously. To minimize the effect of variability between study participants, the run was completed within consecutive days, while carefully controlling for consistent tuning parameters of the mass cytometry instrument (Helios CyTOF, Fluidigm Inc., South San Francisco, CA).

#### 4.2.3 Antibody Staining and Mass Cytometry

The mass cytometry antibody panel included 28 antibodies that were used for phenotyping of immune cell subsets and 11 antibodies for the functional characterization of immune cell responses (**Table S2**). Antibodies were either obtained preconjugated (Fluidigm, Inc.) or were purchased as purified, carrierfree (no BSA, gelatin) versions, which were then conjugated in-house with trivalent metal isotopes utilizing the MaxPAR antibody conjugation kit (Fluidigm, Inc.). After incubation with Fc block (Biolegend), pooled barcoded cells were stained with surface antibodies, then permeabilized with methanol and stained with intracellular antibodies. All antibodies used in the analysis were titrated and validated on samples that were processed identically to the samples used in the study. Barcoded and antibody-stained cells were analyzed on the mass cytometer.

#### 4.2.4 Immune Cell Feature Derivation

The mass cytometry data was normalized using Normalizer v0.1 MATLAB Compiler Runtime (MathWorks) (49). Files were then de-barcoded with a single-cell MATLAB debarcoding tool (50). Manual gating was performed using CellEngine (https://immuneatlas.org/#/) (Primity Bio, Fremont, CA), according to the gating strategy in **Supplementary Figure 3**.

The following cell types were included in the analysis: Granulocytes (CD45^+^CD66^+^), B cells (CD19^+^), NK cells (CD3^−^CD7^+^), CD56^bright^CD16^−^NK, CD56^dim^CD16^+^NK (CD69^−^ and CD69^+^), TCRγδ T cells (CD4^−^CD8^−^), CD4^+^ T cells, CD4T_naive_ (CD45RA^+^CD45RO^−^), CD62L^+^CD4T_naive_, CD4T_effector (eff)_ (CD45RA^+^CD62L^−^), CD4T_memory (mem)_ (CD45RA^−^CD45RO^+^), CD69^+^CD4T_mem_, CD4T_central memory (cm)_ (CD62L^+^CD45RO^+^), CCR5^+^CCR2^+^CD4T_cm_, CD4T_effector memory (em)_ (CD62L^−^CD45RO^+^), CCR5^+^CCR2^+^CD4T_em_, CD25^+^ FoxP3^+^CD4^+^T cells (T_reg_), CD4^+^Tbet^+^T cells (T_h_1), CD8^+^ T cells, CD8T_naive_ (CD45RA^+^CD45RO^−^), CD62L^+^CD8T_naive_, CD8T_eff_ (CD45RA^+^CD62L^−^), CD8T_mem_ (CD45RA^−^CD45RO^+^), CD69^+^CD8T_mem_, CD8T_cm_ (CD62L^+^CD45RO^+^), CCR5^+^CCR2^+^CD8T_cm_, CD8T_em_ (CD62L^−^CD45RO^+^), CCR5^+^CCR2^+^CD8T_em_, NKT cells (CD56^+^CD3^+^), CD4^+^NKT, CD8^+^NKT, TCRγδ NKT, CD14^+^CD16^−^ classical monocytes (cMCs), CD14^−^CD16^+^ non-classical MCs (ncMCs), CD14^+^CD16^+^ intermediate MCs (intMCs), CCR2^+^cMC, CCR2^+^intMC, CCR2^−^ncMC, CD14^+^CD11b^+^HLA-DR^lo^ myeloid-derived suppressor cells (MDSC), CD14^−^CD16^−^HLA-DR^+^ dendritic cells (DC), myeloid DC (CD11c^+^ mDC), HLA-DR^hi^ mDC, HLA-DR^lo^ mDC, and plasmacytoid dendritic cells (CD123^+^ pDC).

#### 4.2.5 Cell Frequency, Basal Intracellular Signaling and Intracellular Signaling Responses

The data from each sample were analyzed for endogenous and intracellular signaling responses in all major adaptive and innate immune cell subsets. Cell frequencies were expressed as a percentage derived from singlet live mononuclear cells (DNA^+^cPARP^−^CD235^−^CD61^−^CD66^−^, except for granulocyte frequencies, which were expressed as percentage of singlet live leukocytes (DNA^+^cPARP^−^CD235^−^CD61^−^). Endogenous intracellular signaling activities at the basal, unstimulated level were quantified per single cell for phosphorylated (p)STAT1, pSTAT3, pSTAT5, pSTAT6, pCREB, pMAPKAPK(pMK)2, pERK1/2, prpS6, pP38, and pNF-κB, and total I-κB using an arcsinh transformed value calculated from the median signal intensity. Intracellular signaling responses to stimulation were reported as the difference in arcsinh transformed value of each signaling protein between the stimulated and unstimulated conditions (arcsinh ratio over endogenous signal). A knowledge-based penalization matrix was applied to intracellular signaling response features in the mass cytometry data based on mechanistic immunological knowledge, as previously described (24, 51). Importantly, mechanistic priors used in the penalization matrix are independent of immunological knowledge related to pregnancy or the evolution of labor.

### 4.3 Statistical Analyses

#### 4.3.1 Multivariate Modeling

For the multivariate analysis, we trained a LASSO model on each sample using the time since induction as a predictor. The L1 regularization is used to increase model sparsity for the sake of biological interpretation and model robustness. For the given *n* × *p* matrix *X* of *p* biological features derived from each of *n* samples, and given the vector of predictor variables *y* = (*y*_1_, … ,*y_n_*), we fit the regression coefficients $\hat{\beta }=({\hat{\beta }}_{1},\ldots ,{\hat{\beta }}_{p})$ such that $\hat{\beta }=\underset{\beta }{\arg\min}\left({\left||y-X\beta |\right|}^{2}+\lambda {\left||\beta |\right|}_{1}\right)$. The optimal γ is identified during the cross-validation procedure.

#### 4.3.2 Cross-Validation

To take into account the dependency of samples coming from the same patient at different timepoints, we evaluated our pipeline using a leave-one-patient-out cross-validation. For each iteration of the cross-validation, we single out all the samples coming from one patient and train a model on the rest. We then predict the samples from the one blinded patient. The predictions are then combined and give the estimate of performance of the model on all the patients. We report model results with the Pearson R coefficient and the p-value associated.

#### 4.3.3 Correlation Network and Heatmap

The results are visualized with a correlation network computed from the correlation matrix of all the features. The features are mapped using a t-distributed stochastic neighbor embedding (tSNE) layout in a graph-fashion (52) using the correlation coefficients as edge weight for correlation coefficients above 0.9. We used this representation to show the interaction between the immune features and we also report the result of the modeling on this representation, with different node size and colors.

We also used a heatmap representation of the perturbations happening at the different timepoints compared to baseline. To compute the heatmap, we z-score transformed the difference between the median of a feature at a given timepoint and the baseline median. We then only plotted the differences that passed Wilcoxon signed-rank test with a p-value less than 0.05 at one of the timepoints. We plotted the remaining features to observe the trajectories.

#### 4.3.4 Bootstrap Analysis and Comparison of Ranking

We combined our multivariate model fitting on our high-dimensional immunome dataset with a bootstrap analysis where we repeat a random sampling with replacement on the dataset and train a cross-validated model each time. At each iteration, we keep the non-zero coefficients selected by the LASSO model on the bootstrapped dataset and we repeat the procedure 1,000 times. We report the frequency of selection of the features (number of times the feature occurs in the model), their p-value to be more selected than an artificial noisy feature, and the ratio over the most frequently selected decoy feature in all bootstraps. To generate the artificial noisy feature, we operate by random permutation of the original immune features in the dataset. Permutation is repeated at each iteration of the bootstrap, allowing for an estimate of the noise picked up by the LASSO model. To assess the relative importance of each feature to the model, we ranked features in the dataset based on their frequency of selection over the decoy features.

## Data Availability Statement

The dataset presented in this study can be accessed online in the Dryad repository under https://doi.org/10.5061/dryad.80gb5mkrg.

## Ethics Statement

The studies involving human participants were reviewed and approved by Institutional Review Board, Stanford University, School of Medicine (Approval ID: 44576). The patients/participants provided their written informed consent to participate in this study.

## Author Contributions

Conceptualization: KA, BC, and BG. Methodology: KA, JH, EG, and IS. Software: JH. Validation: KA, JH, EG, and IS. Formal analysis: KA, JH, and IS. Investigation: KA, JH, IS, DF, XH, EG, ET, LP, FV, and AT. Resources: NA, MA, YB, PS, BC, and BG. Data Curation: KA, JH, and IS. Writing - Original Draft: KA, JH, IS, and BG. Writing - Review & Editing: KA, JH, DF, XH, ET, IM, RW, MA, YB, PS, BC, IS, BG, and all authors. Visualization: KA, JH, and IS. Supervision: BC and BG. Project administration: BC, DS, and BG. Funding acquisition: BC and BG. All authors contributed to the article and approved the submitted version.

## Funding

The study was supported in part by the Doris Duke Charitable Foundation (to BG); the German Research Foundation (STE2757/1-1, to IS); the Stanford Maternal and Child Health Research Institute Grant & Postdoctoral Award (to XH, LP); the Prematurity Research Fund; the March of Dimes Prematurity Research Center at Stanford University (#22FY19343); the Bill & Melinda Gates Foundation (OPP1189911), and the Center for Human Systems Immunology pilot seed grant (to BG); the Stanford Maternal and Child Health Research Institute (to DS, RW, BG, NA, MA); the Charles and Mary Robertson Foundation (to DF, XH, LP, XH, NA, DS); the NIH [R35GM137936 (to BG), R35 GM138353 (to NA)]; the Burroughs Wellcome Fund (BMGF OPP1113682, to NA). PS is an Arline and Pete Harman Endowed Faculty Scholar of the Stanford Maternal and Child Health Research Institute.

## Conflict of Interest

The authors declare that the research was conducted in the absence of any commercial or financial relationships that could be construed as a potential conflict of interest.

## Publisher’s Note

All claims expressed in this article are solely those of the authors and do not necessarily represent those of their affiliated organizations, or those of the publisher, the editors and the reviewers. Any product that may be evaluated in this article, or claim that may be made by its manufacturer, is not guaranteed or endorsed by the publisher.
