# Supplementary material for: A Peripheral Immune Signature of Labor Induction
Source: Front Immunol. 2021 Sep 9;12:725989. doi: 10.3389/fimmu.2021.725989 (PMC8458888; doi:10.3389/fimmu.2021.725989)
Supplement: Supplementary file 1 [file DataSheet_1.pdf]

Supplementary Material

doi: 10.3389/fimmu.2021.725989

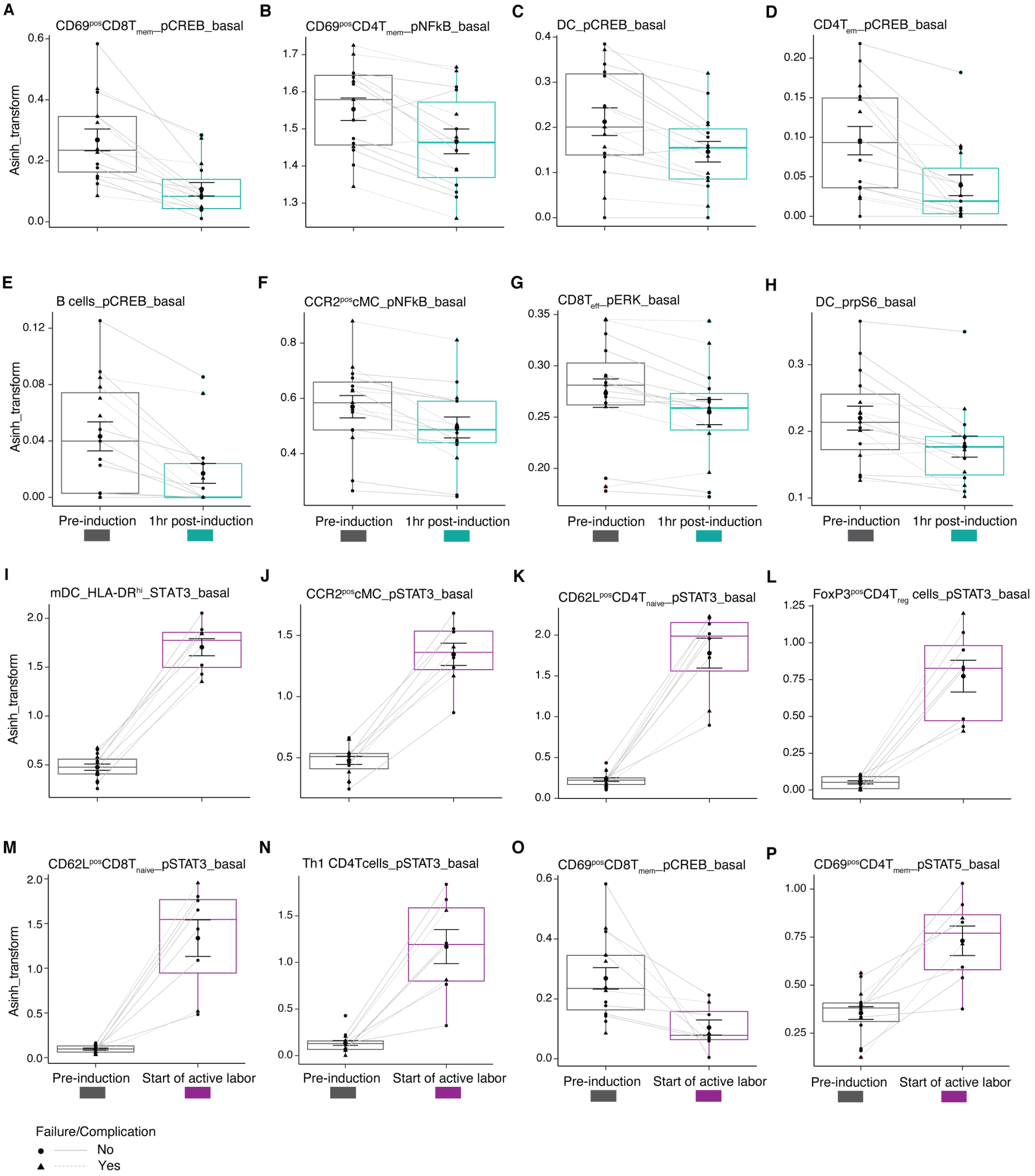

Supplementary Figure 1. Immune modulation 1 hr after induction (T2, teal) and at start of active labor (T5, purple) in comparison to pre-induction (baseline, T1, gray) (N = 15, n = 48). Shown are representative features. (**A–H**) Features significantly different ($p < 0.05$, Wilcoxon signed-rank test for T1 vs. T2 or rank-sum test for T1 vs. T3-5) in univariate paired comparisons. (**I–P**) Features

significantly different ($p < 0.05$) in univariate unpaired comparisons. Related to Fig. 2 and Supplementary Table 2.

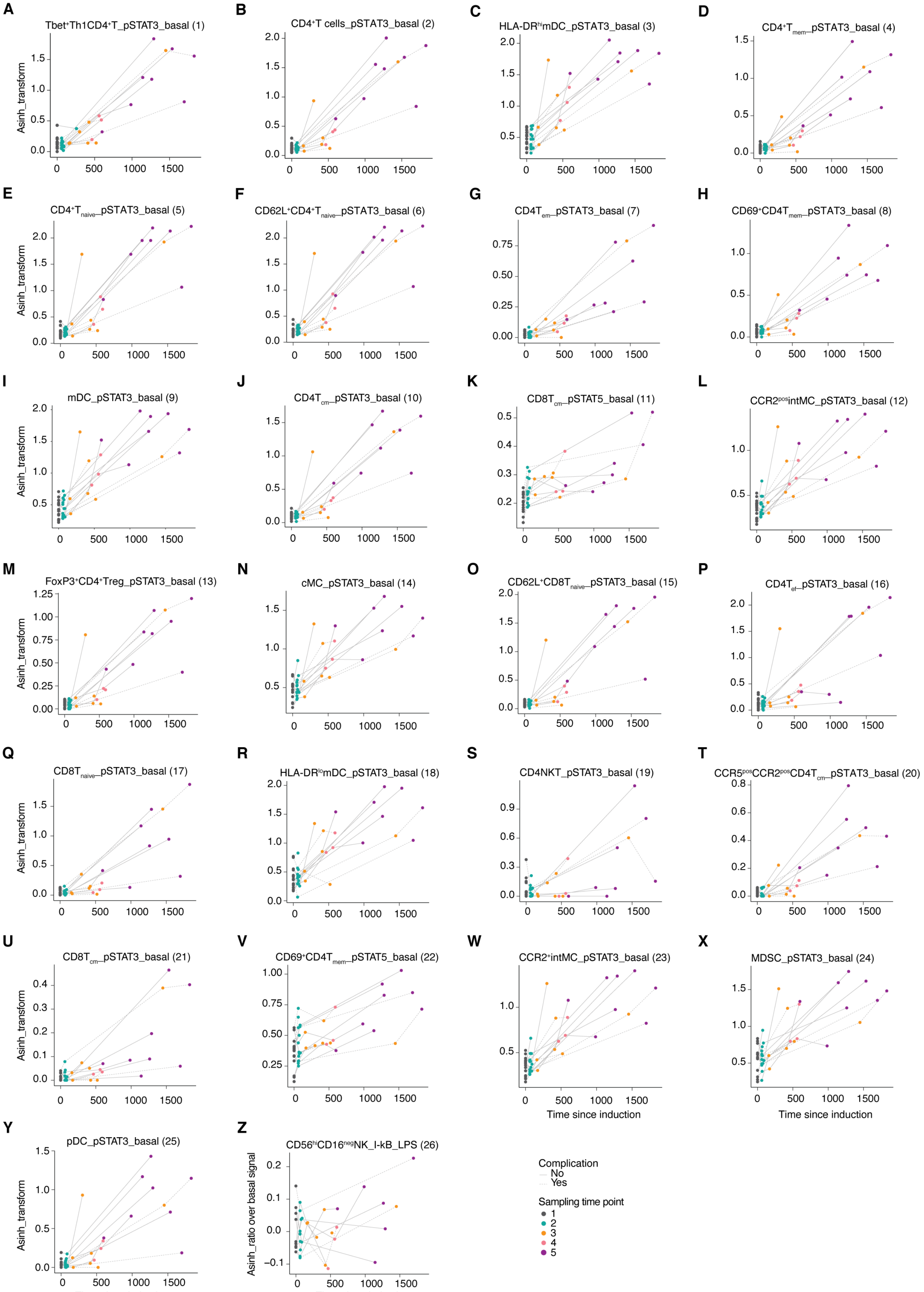
A
Tbet+Th1CD4+T_pSTAT3_basal (1)
B
CD4+T cells_pSTAT3_basal (2)
C
HLA-DRhimDC_pSTAT3_basal (3)
D
CD4+Tmem_pSTAT3_basal (4)
E
CD4+Tnaive_pSTAT3_basal (5)
F
CD62L+CD4+Tnaive_pSTAT3_basal (6)
G
CD4Tem_pSTAT3_basal (7)
H
CD69+CD4Tmem_pSTAT3_basal (8)
I
mDC_pSTAT3_basal (9)
J
CD4Tcm_pSTAT3_basal (10)
K
CD8Tcm_pSTAT5_basal (11)
L
CCR2posintMC_pSTAT3_basal (12)
M
FoxP3+CD4+Treg_pSTAT3_basal (13)
N
cMC_pSTAT3_basal (14)
O
CD62L+CD8Tnaive_pSTAT3_basal (15)
P
CD4Tef_pSTAT3_basal (16)
Q
CD8Tnaive_pSTAT3_basal (17)
R
HLA-DRlomDC_pSTAT3_basal (18)
S
CD4NKT_pSTAT3_basal (19)
T
CCR5posCCR2posCD4Tcm_pSTAT3_basal (20)
U
CD8Tcm_pSTAT3_basal (21)
V
CD69+CD4Tmem_pSTAT5_basal (22)
W
CCR2+intMC_pSTAT3_basal (23)
X
MDSC_pSTAT3_basal (24)
Y
pDC_pSTAT3_basal (25)
Z
CD56hiCD16negNK_I-kB_LPS (26)
Asinh_transform
Asinh_ratio over basal signal
Time since induction
Complication
No
Yes
Sampling time point
1
2
3
4
5

Supplementary Figure 2. Top features most informative for the prediction model (N = 15, n = 48). Number (1-26) indicates feature rank based on occurrence in a bootstrap model. Related to Fig. 3.

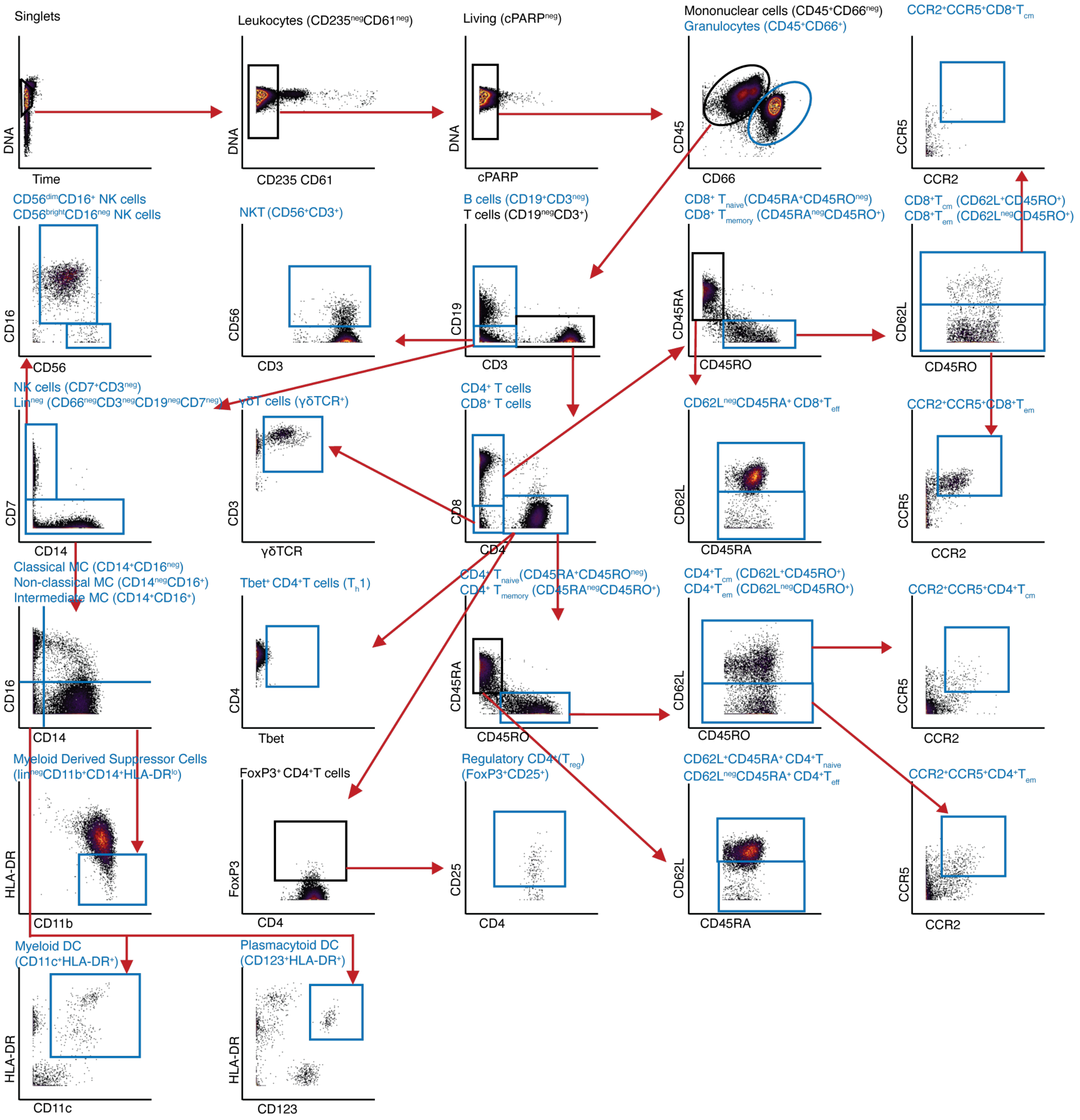

Supplementary Figure 3. Gating strategy for mass cytometry analyses. Live, non-erythroid cell populations (blue subpopulations) were used for analysis.

Antibody	Manufacturer	Atomic Symbol	Atomic Mass	Clone	Comment	Catalogue Number	RRID
Barcode 1	Trace Sciences	Pd	102		Barcode		
Barcode 2	Trace Sciences	Pd	104		Barcode		
Barcode 3	Trace Sciences	Pd	105		Barcode		
Barcode 4	Trace Sciences	Pd	106		Barcode		
Barcode 5	Trace Sciences	Pd	108		Barcode		
Barcode 6	Trace Sciences	Pd	110		Barcode		
CD235	BioLegend	In	113	HIR2	Phenotype	306615	AB_2562825
CD61	BD	In	113	VI-PL2	Phenotype	555752	AB_396093
CD45	Biolegend	In	115	HI30	Phenotype	304045	AB_2562821
CD66	BD	La	139	B1.1/CD66	Phenotype	551354	AB_394166
CD7	BD	Pr	141	M-T701	Phenotype	555359	AB_395762
CD19	BioLegend	Nd	142	HIB19	Phenotype	302247	AB_2562815
CD45RA	BioLegend	Nd	143	HI100	Phenotype	304143	AB_2562822
CD11b	BioLegend	Nd	144	ICRF44	Phenotype	301337	AB_2562811
CD4	BioLegend	Nd	145	RPA-T4	Phenotype	300541	AB_2562809
CD8a	BioLegend	Nd	146	RPA-T8	Phenotype	301053	AB_2562810
CD11c	BioLegend	Sm	147	Bu15	Phenotype	337221	AB_2562834
CD123	BioLegend	Nd	148	6H6	Phenotype	306027	AB_2562823
pCREB (pS133)	CST	Sm	149	87G3	Function	9198	AB_2561044
pSTAT5 (Tyr694)	CST	Nd	150	C11C5	Function	9359	AB_823649
pP38 (pT180/pY182)	BD	Eu	151	36/p38	Function	612289	AB_399606
TCRγδ	BD	Sm	152	B1	Phenotype	555715	AB_396059
pSTAT1 (pY701)	BD	Eu	153	14/P-STAT1	Function	612133	AB_399504
pSTAT3 (Tyr705)	CST	Sm	154	M9C6	Function	4113	AB_2198588
pS6 (pS235/pS236)	CST	Gd	155	D57.2.2E	Function	4858	AB_916156
IκB	CST	Gd	156	L35A5	Function	4814S	
CD69	BD	Gd	157	FN50	Phenotype	555529	AB_395914
CD33	BioLegend	Gd	158	WM53	Phenotype	303419	AB_2562818
pMPK2 (Thr334)	CST	Tb	159	27B7	Function	3007	AB_490936
Tbet	Thermo Fisher	Gd	160	4B10	Phenotype	14582580	AB_763635
cPARP	BD	Dy	161	F21-852	Function	552596	AB_394437
FoxP3	Thermo Fisher	Dy	162	PCH101	Phenotype	14477682	AB_467554
CD45RO	Fluidigm	Dy	164	UCHL1	Phenotype	3164007B	AB_2811092
CD16	BioLegend	Ho	165	3G8	Phenotype	302051	AB_2562814
pNFκB (pS529)	BD	Er	166	k108951250	Function	558393	AB_647284
pERK1/2 (pT202/pY204)	CST	Yb	167	D13.14.4E	Function	4370	AB_2315112
pSTAT6 (Tyr641)	BioLegend	Er	168	A15137E	Function	686002	AB_2616820
CD25	BioLegend	Tm	169	M-A251	Phenotype	356102	AB_2561752
CD3	BioLegend	Er	170	UCHT1	Phenotype	300402	AB_314056
CCR5	Fluidigm	Yb	171	NP6G4	Phenotype	3171017a	
CD62L	Thermo Scientific	Yb	172	DREG.200	Phenotype	BMS1015	AB_10596353
CCR2	BioLegend	Yb	173	K036C2	Phenotype	357202	AB_2561851
HLA-DR	BioLegend	Yb	174	L243	Phenotype	307651	AB_2562826
CD14	BioLegend	Yb	175	M5E2	Phenotype	301843	AB_2562813
CD56	BD	Yb	176	NCAM16.2	Phenotype	559043	AB_397180
DNA1/2	Fluidigm	Ir	191/192		DNA		

Supplementary Table 1. Mass cytometry antibody panel.

T2 1 hr post-induction vs. T1 baseline	Feature	p (paired)	p adj	FC
1	CD69posCD8Tmem_pCREB_basal	3.51E-05	0.0241	-0.151
2	CD8Tmem_pCREB_basal	8.70E-05	0.0246	-0.100
3	CD8Tem_pCREB_basal	0.00011	0.0246	-0.117
4	CD8Tcells_pCREB_basal	0.00031	0.0466	-0.075
5	CCR5posCCR2posCD8Tem_pCREB_basal	0.00052	0.0466	-0.155
6	CD69posCD4Tmem_pNF-κB_basal	0.00056	0.0466	-0.116
7	CD8Tef_pCREB_basal	0.00067	0.0466	-0.087
8	DCs_pCREB_basal	0.00071	0.0466	-0.046
9	CD4Tem_pCREB_basal	0.00077	0.0466	-0.074
10	Bcells_pCREB_basal	0.00084	0.0466	-0.040
11	TCRgdposNKT_pCREB_basal	0.00084	0.0466	-0.113
12	CCR2poscMCs_pNF-κB_basal	0.00086	0.0466	-0.097
13	CD8Tef_pERK_basal	0.00091	0.0466	-0.022
14	cMCs_pNF-κB_basal	0.00100	0.0466	-0.102
15	CCR5posCCR2posCD8Tem_prpS6_basal	0.00107	0.0466	-0.022
16	CD8Tcm_pCREB_basal	0.00111	0.0466	-0.077
17	CCR5posCCR2posCD4Tem_pCREB_basal	0.00121	0.0466	-0.065
18	DCs_prpS6_basal	0.00128	0.0466	-0.037
19	TCRgdposT_pCREB_basal	0.00130	0.0466	-0.108
20	MDSCs_pNF-κB_basal	0.00139	0.0466	-0.088
21	CD62LposCD8Tnaive_pCREB_basal	0.00143	0.0466	-0.036
**T3 Regular contractions vs. T1 baseline**	**Feature**	**p (unpaired)**	**p adj**	**FC**
1	TCRgdposT_pCREB_basal	0.00020	0.0807	-0.121
2	TCRgdposNKT_pCREB_basal	0.00024	0.0807	-0.131
3	CD8Tcm_pSTAT5_basal	0.00050	0.0809	0.082
4	CD8Tcells_pCREB_basal	0.00063	0.0809	-0.078
5	CD8Tef_pCREB_basal	0.00074	0.0809	-0.095
6	CCR5posCCR2posCD8Tem_pCREB_basal	0.00103	0.0809	-0.151
7	CD4Tem_pCREB_basal	0.00115	0.0809	-0.078
8	CD8Tmem_pCREB_basal	0.00126	0.0809	-0.095
9	CD69posCD8Tmem_pCREB_basal	0.00127	0.0809	-0.135
10	CCR5posCCR2posCD4Tem_pCREB_basal	0.00127	0.0809	-0.070
11	CD8Tem_pCREB_basal	0.00130	0.0809	-0.109
12	CD69negCD56loCD16posNK_pCREB_basal	0.00190	0.0967	-0.105

13	CD56loCD16posNK_pCREB_basal	0.00191	0.0967	-0.105
14	CD8Tmem_pSTAT5_basal	0.00198	0.0967	0.049
15	NK_pCREB_basal	0.00213	0.0974	-0.101
**T5 Start of active labor vs. T1 baseline**	**Feature**	**p (unpaired)**	**p adj**	**FC**
1	mDCsHLADRhi_pSTAT3_basal	3.63E-07	0.0002	1.297
2	mDCs_pSTAT3_basal	3.11E-06	0.0011	1.245
3	CCR2poscMCs_pSTAT3_basal	9.72E-06	0.0018	0.851
4	cMCs_pSTAT3_basal	1.06E-05	0.0018	0.837
5	mDCsHLADRlo_pSTAT3_basal	1.66E-05	0.0023	1.202
6	CCR2posintMCs_pSTAT3_basal	5.07E-05	0.0049	0.766
7	CD62LposCD4Tnaive_pSTAT3_basal	5.74E-05	0.0049	1.763
8	MDSCs_pSTAT3_basal	5.12E-05	0.0049	0.837
9	CD4posTnaive_pSTAT3_basal	6.80E-05	0.0052	1.736
10	intMCs_pSTAT3_basal	0.00012	0.0085	0.661
11	CD4Tcells_pSTAT3_basal	0.00021	0.0119	1.400
12	CD4Tcm_pSTAT3_basal	0.00020	0.0119	1.147
13	Tregs_pSTAT3_basal	0.00026	0.0139	0.775
14	CD69posCD4Tmem_pSTAT3_basal	0.00044	0.0216	0.683
15	CD62LposCD8Tnaive_pSTAT3_basal	0.00050	0.0226	1.451
16	CCR5posCCR2posCD4Tem_frequency_basal	0.00053	0.0226	-0.638
17	CD4Tmem_pSTAT3_basal	0.00065	0.0264	0.800
18	Th1_pSTAT3_basal	0.00070	0.0267	1.064
19	CD69posCD8Tmem_pCREB_basal	0.00118	0.0404	-0.156
20	pDCs_pSTAT3_basal	0.00113	0.0404	0.831
21	CD69posCD4Tmem_pSTAT5_basal	0.00125	0.0407	0.389
22	Granulocytes_pSTAT3_basal	0.00132	0.0412	0.362
23	CCR5posCCR2posCD4Tcm_pSTAT3_basal	0.00158	0.0471	0.383

Supplementary Table 2. Univariate comparisons of timepoints during the latent phase of labor (T2, T3, and T5) vs. pre-induction baseline (T1). Features with FDR-adjusted $p < 0.05$ were considered significantly different. Fold-change (FC) indicates directionality of change vs. baseline. Related to Fig. 2 and Supplementary Figure 1.

Feature Rank	Feature	Occurrence in 1000 model iterations	Variance	Ratio over most frequent decoy feature	p associated with complications	Coeff - EN
1	Th1_ pSTAT3_basal	98.31	1.34	10.35	0.21	418.1
2	CD4Tcells_pSTAT3_basal	97.87	1.66	10.30	0.07	0.0
3	mDCsHLADRhi_pSTAT3_ basal	92.09	2.86	9.69	0.10	35.6
4	CD4Tmem_pSTAT3_basal	90.24	4.20	9.50	0.21	0.0
5	CD4posTnaive_pSTAT3_basal	86.01	3.79	9.05	0.02	3.6
6	CD62LposCD4Tnaive_pSTAT3 _basal	85.44	3.76	8.99	0.02	0.0
7	CD4Tem_pSTAT3_basal	83.04	3.95	8.74	0.93	0.0
8	CD69posCD4Tmem_pSTAT3_ basal	82.22	4.45	8.65	0.18	0.0
9	mDCs_pSTAT3_basal	79.31	4.90	8.35	0.15	350.3
10	CD4Tcm_pSTAT3_basal	70.72	8.78	7.44	0.06	0.0
11	CD8Tcm_pSTAT5_basal	67.22	8.03	7.08	0.84	222.2
12	CCR2poscMCs_pSTAT3_basal	47.77	5.67	5.03	0.28	0.0
13	Tregs_pSTAT3_basal	47.51	5.87	5.00	0.12	0.0
14	cMCs_pSTAT3_basal	44.53	5.55	4.69	0.30	0.0
15	CD62LposCD8Tnaive_pSTAT3 _basal	36.82	5.35	3.88	0.07	0.0
16	CD4Tef_pSTAT3_basal	34.64	4.73	3.65	0.64	66.7
17	CD8Tnaive_pSTAT3_basal	29.28	4.47	3.08	0.81	0.0
18	mDCsHLADRlo_pSTAT3_ basal	28.23	6.19	2.97	0.26	1.7
19	CD4NKT_pSTAT3_basal	26.88	5.85	2.83	0.74	0.0
20	CCR5posCCR2posCD4Tcm_ pSTAT3_basal	25.17	4.48	2.65	0.11	0.0
21	CD8Tcm_pSTAT3_basal	25.04	6.15	2.64	0.94	194.9
22	CD69posCD4Tmem_pSTAT5_b asal	20.98	5.12	2.21	0.93	0.0
23	CCR2posintMCs_pSTAT3_ basal	19.64	4.06	2.07	0.31	0.0
24	MDSCs_pSTAT3_basal	14.04	3.80	1.48	0.72	0.0
25	pDCs_pSTAT3_basal	14.04	3.58	1.48	0.09	0.0
26	CD56hiCD16negNK_I-κB_LPS	13.83	8.48	1.46	0.91	0.0
	Decoy196	9.50	16.15			

Supplementary Table 3. Top informative features of the model predicting time since induction. Features were ranked based on number of bootstrap occurrences. Related to Fig. 3 and 4.
